# Comparison of the Activity of Fecal Enzymes and Concentration of SCFA in Healthy and Overweight Children

**DOI:** 10.3390/nu15040987

**Published:** 2023-02-16

**Authors:** Katarzyna Śliżewska, Michał Włodarczyk, Martyna Sobczak, Renata Barczyńska, Janusz Kapuśniak, Piotr Socha, Aldona Wierzbicka-Rucińska, Aneta Kotowska

**Affiliations:** 1 Institute of Fermentation Technology and Microbiology, Department of Biotechnology and Food Sciences, Technical University of Lodz, Wolczanska 171/173, 90-924 Łódź, Poland; 2 Department of Dietetics and Food Studies, Faculty of Science and Technology, Jan Dlugosz University, Armii Krajowej 13/15, 42-200 Czestochowa, Poland; 3 The Children’s Memorial Health Institute, aleja Dzieci Polskich 20, 04-736 Warsaw, Poland

**Keywords:** obesity, SCFA, BCFA, bacterial enzymes, gut microbiota

## Abstract

In modern societies obesity has become a serious issue which must be urgently addressed. The health implications of neglected obesity are substantial, as not only does it affect individuals’ everyday lives, but it also leads to significantly increased mortality due to the development of several disorders such as type-2 diabetes, cardiovascular diseases, cancers, and depression. The objective of this research was to investigate the alterations in selected health markers caused by overweight and obesity in children. The measured parameters were the activity of the fecal enzymes, the concentration of short-chain fatty acids (SCFAs), and the concentration of branched-chain fatty acids (BCFAs). The activity of the fecal enzymes, specifically α-glucosidase, α-galactosidase, β-glucosidase, β-galactosidase, and β-glucuronidase, was determined using spectrophotometry at a wavelength of 400 nm. Furthermore, concentrations of lactic acid, SCFAs (formic, acetic, propionic, butyric, and valeric acids), and BCFAs (isobutyric and isovaleric acids) were determined using the HPLC method. The obtained results reveal that obese children have different fecal enzyme activity and a different profile of fatty acids from children of normal weight. The group of obese children, when compared to children of normal weight, had increased concentrations of BCFAs (*p* < 0.05) and higher activity of potentially harmful enzymes such as β-glucosidase and β-glucuronidase (*p* < 0.05). In comparison, children of normal weight exhibited significantly increased concentrations of lactic acid and SCFAs (especially formic and butyric acids) (*p* < 0.05). Furthermore, their α-glucosidase and α-galactosidase activity were higher when compared to the group of obese children (*p* < 0.05). These results suggest that the prevalence of obesity has a significant impact on metabolites produced in the gastrointestinal tract, which might result in a higher chance of developing serious diseases.

## 1. Introduction

Obesity has become an epidemic of the 21st century, which is confirmed by the alarming data from the WHO on the NCD-RisC indicating a rapid increase in the prevalence of metabolic disorders in well-developed countries [1,2]. Records show that in 2016 more than 1.9 billion adults were overweight, of whom 13% were obese, meaning they had a BMI higher than 30 [3]. Similar findings concern children and adolescents. It was estimated by the WHO that over 340 million children aged 5–19 years were overweight in 2016, and over 30% of these were obese [4]. This is alarming given the fact that obese children have a significantly higher chance of becoming obese adults than non-obese children, which will directly impact the number of people suffering from obesity-related health implications [5]. Since then, there has been no record of improvement in the situation. According to several predictions, by the year 2030 the number of obese children may double and further increase with time [2,6,7].

There are various factors that promote the development of metabolic disorders, such as lifestyle changes that favor sitting or less active occupations, high stress levels, rush, and ill-considered dietary decisions [8]. It is well-established knowledge that diet influences the gastrointestinal microbiota, which is reflected in several studies, i.e., comparing the microbiota of normal-weight and obese people or examining how certain types of food impact the gut microorganisms [9,10,11,12,13]. Apart from contributing to energy metabolism, gut microorganisms produce a variety of bioactive compounds, such as vitamins, short-chain and branched-chain fatty acids (SCFAs, BCFAs), and fecal enzymes, which participate in several metabolic pathways where they exhibit anti-inflammatory, anticarcinogenic, and antioxidative effects (genera Lactobacillus, Bifidobacterium) [14,15,16]. In contrast, a dysbiosis of gut microorganisms, or their low diversity favoring the domination of certain genera (Bacteroides, Clostridium, Escherichia, Enterococcus), may cause reduced synthesis of bioactive compounds and promote synthesis with the possible accumulation of potentially harmful ones [17,18].

SCFAs have a variety of beneficial effects on the host organism, i.e., regulation of energy metabolism, immunoregulation, and stabilization of the integrity of the intestinal barrier [8,19]. BCFAs, however, are often associated with the process of protein fermentation, which may lead to the accumulation of potentially harmful bioactive compounds and can increase the risk of developing colonic cancer [20]. Microbial diversity in the gastrointestinal tract is similarly a factor that influences enzymatic activity in the colon. Bacteria produce a variety of enzymes that help to digest food ingredients while producing several bioactive compounds as byproducts. Reductases and hydrolases, which are the main classes of enzymes secreted by the intestinal bacteria, can help to produce beneficial SCFAs, but may likewise be responsible for the formation of toxic or carcinogenic compounds [21,22]. Due to the high impact of the above-mentioned bioactive compounds on the human organism, they can be considered health markers, which help to assess the physical status of a person.

Based on these facts, the aim of this study was to further confirm differences in the activities of fecal enzymes and the profiles of fatty acids in fecal samples between a group of healthy overweight or obese children and a group of children of normal weight. Accordingly, concentrations of SCFAs, namely formic, acetic, propionic, butyric, and valeric acids, together with concentrations of lactic acid were determined using the HPLC method. Furthermore, concentrations of BCFAs such as isobutyric and isovaleric acids were measured. The next part of the study involved the determination of activity of fecal enzymes, namely α-glucosidase, α-galactosidase, β-glucosidase, β-galactosidase, and β-glucuronidase, in the same fecal samples. The outcome of the experimental work allowed for the evaluation whether the group of obese individuals without dietary supervision has significantly different health markers (fecal enzyme activity and concentration of fatty acids). The obtained results were subject to statistical analysis to verify possible correlations among the mentioned health markers and elucidate more comprehensive conclusions.

## 2. Materials and Methods

### 2.1. Biological Material

The fecal samples were collected from 97 overweight and obese children (participants of the PreSTFibre4kids research program) and 26 children of normal weight (both males and females) aged 6–10 years (Table 1). Patients from the PreSTFibre4kids program were children with one of the following health implications: simple obesity and overweight without organ complications, fatty liver disease, and high blood pressure. Volunteers were recruited in Poland. Every participant had voluntarily signed a consent form that confirmed his/her involvement in the project.

Exclusion criteria from the study included organ failure, metformin treatment, and allergy to any component of the prebiotic preparation that would be used in further phases of the research. The exclusion criteria did not include a medical history of taking antibiotics or the usage of dietary supplements. The overweight status of children was assigned according to the WHO’s BMI classification charts [23].

Participants were given general dietary advice by a trained professional.

Fecal samples were transferred into sterile containers and frozen immediately (−20 °C) prior to transport to the laboratory.

The project was accepted by the Research Ethics Committee of the Children’s Memorial Health Institute in Warsaw, Poland (18/KBE/2021).

### 2.2. Activity of Fecal Enzymes

Prior to the enzymatic assays, the samples were prepared according to the following protocol. Firstly, 0.7 g of sample was suspended in 3.5 mL of 0.2 M phosphate buffer and vortexed thoroughly (Vortex RS-VA 10, Phoenix Instrument, Garbsen, Germany). Secondly, the samples underwent sonification (Time = 2 min, Amplitude = 60, Pulse = 6 s, Cole–Parmer Instrument Co., Vernon Hills, IL, USA). Afterwards, the samples were centrifuged (12,000 rpm, Time = 20 min, Centrifuge MPW-251, MPW, Warszawa, Poland) and the supernatant was transferred to the sterile Eppendorf tubes.

With the use of spectrophotometric methods, the activity of the fecal enzymes α-glucosidase, α-galactosidase, β-glucosidase, β-galactosidase, and β-glucuronidase was determined. The protocols used in the study were based on the reaction of α-glucosidase, β-glucosidase, α-galactosidase, β-galactosidase, and β-glucuronidase with 4-nitrophenyl α-D-glucopyranoside (TCI, Tokyo, Japan), 4-nitrophenyl β-D-glucopyranoside (TCI, Tokyo, Japan), 4-nitrophenyl α-D-galactopyranoside (TCI, Tokyo, Japan), 4-nitrophenyl β-D-galactopyranoside (TCI, Tokyo, Japan), and 4-nitrophenyl β-D-glucuronide (TCI, Tokyo, Japan), respectively.

The used substrates were specific to the respective enzymes present in the fecal sample. The reaction mixture contained 0.5 mL of phosphate buffer (pH = 7, 0.02 M), 0.05 mL of substrate solution (20 mM), and 0.25 mL of sample. Incubation was performed at 37 °C for 15 min (α-glucosidase, α-galactosidase and β-glucuronidase) or 60 min (β-glucosidase and β-galactosidase).

An observed hue shift in the sample to yellow proved the reaction had taken place. The intensity of the color was directly proportional to the amount of p-nitrophenol released. The reactions were inhibited using 0.25 M sodium carbonate after the given reaction time. The absorbance of the samples was measured using the spectrophotometer Rayleigh UV-2601 (BFRL, Beijing, China) at a wavelength of λ = 400 nm. The unit of enzyme activity refers to the amount of p-nitrophenol (expressed in µM) which was released during 1 h of reaction for 1 mg of protein in 1 mL of sample [µMh·mg^−1^].

### 2.3. Concentration of Lactic Acid, SCFAs and BCFAs

Prior to the HPLC analysis the samples were prepared according to the following protocol. Firstly, 0.5 g of sample was suspended in 3 mL of demineralized water and vortexed thoroughly (Vortex RS-VA 10, Phoenix Instrument, Garbsen, Germany). Afterwards, samples were centrifuged (12,000 rpm, Time = 20 min, Centrifuge MPW-251, MPW, Poland) and the supernatant was filtered (0.22 µm filters, ALWSCI Technologies, Shaoxing, China) and transferred to the sterile autosampler vials.

HPLC with the Surveyor liquid chromatography system (Termo Scientifc, Waltham, MA, USA) was employed in the experiment. The following parameters of the process were used: Aminex HPX-87H column (300 × 7.8 mm), UV detector, 0.005 mL^−1^ sulphuric acid as eluent, flow rate 0.6 μLmin^−1^, single sample analysis time 40 min.

### 2.4. Statistical Analysis

The normality of the distribution of variables was examined with the Shapiro–Wilk test, whereas the homogeneity of variances was assessed with Bartlett’s test. After the confirmation of normality and equal variance, the results were analyzed using the one-way ANOVA test and Tukey’s post hoc test. Python was used for statistical testing, where the *p*-value of 0.05 was considered significant. The data is presented in a mean ± standard deviation (SD) format.

## 3. Results

### 3.1. Analysis of Metabolites

The main objective of the analysis was to determine the influence of excess weight on the concentrations of lactic acid, SCFAs, and BCFAs, as described in the previous section. The primary hypothesis was that the obese subjects would have altered concentrations of fatty acids due to the alterations in intestinal microbiota commonly seen in such patients. Specifically, it was suspected that the concentrations of lactic acid and SCFAs (formic, acetic, propionic, butyric, and valeric acids) would be lower in obese patients, while the concentrations of BCFAs (isobutyric and isovaleric acids) might be higher.

**Figure 1:**
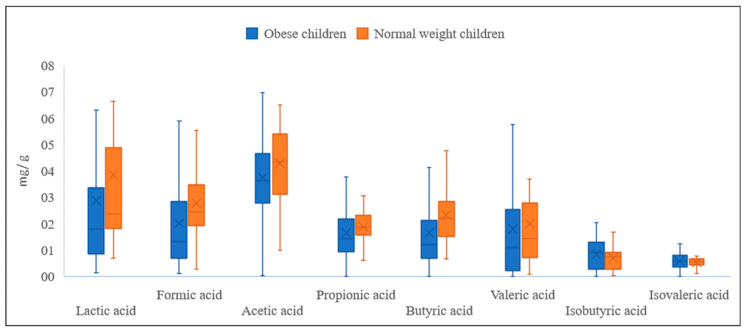
Concentrations of lactic acid, SCFAs, and BCFAs, in obese and normal-weight children.

It was determined that the concentrations of lactic acid and most of the SCFAs were significantly higher (*p* < 0.05) in the group of children of normal weight, whereas the concentration of BCFAs was lower (Figure 1). In the case of lactic acid, the concentration in the obese subjects was 24.9% lower in comparison to the children of normal weight (Table 2). Similarly, the concentrations of formic and butyric acids were lower by 27.3% and 29.0%, respectively, which was a clear confirmation of the hypothesis. The concentrations of acetic, propionic, and valeric acids also maintained that trend, as they were lower in the obese children by, respectively, 12.7%, 12.2%, and 10.1%. Conversely, the concentration of isobutyric acid was noticeably elevated in the group of obese children (15.3% higher in comparison to the children of normal weight). In the case of isovaleric acid, the difference in concentration was not significant; however, it was slightly higher in the group of obese children (Table 2).

In addition to the main objective of the study, the differences in fatty acid profiles were also assessed between male and female participants to evaluate whether sex could possibly have an influence. According to the obtained results for the total population of study participants, the differences in concentrations between the tested groups for lactic, formic, and isobutyric acids were statistically significant (*p* < 0.05). As portrayed in Table 3, the concentration of lactic and valeric acids were significantly higher in the case of males, by 24.3% and 35.1% (*p* < 0.05), respectively. On the contrary, the concentrations of formic, isobutyric, and isovaleric acids were lower in the male group by 18.5%, 21.4% and 19.1% (*p* < 0.05), respectively. In the case of acetic, propionic, and butyric acids, the concentrations were not significantly different between groups.

**Figure 2:**
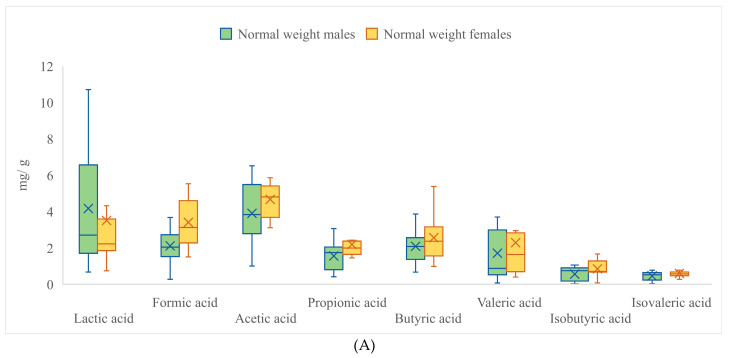

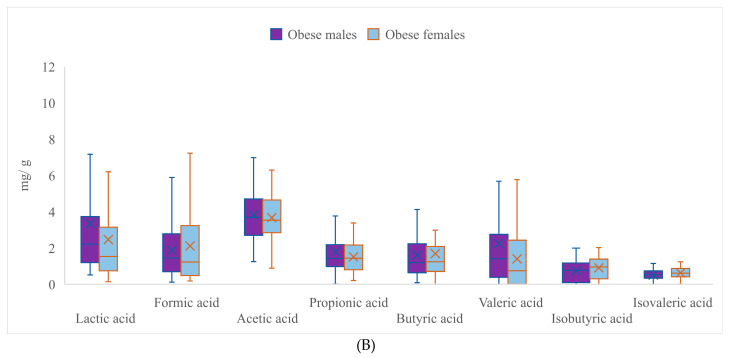
Concentrations of lactic acid, SCFAs, and BCFAs differentiated between males and females and according to anthropometric status (**A**)—presents data of normal weight males and females; (**B**)—presents data of obese males and females.

When the comparison was made within the group of children of normal weight and with obese children, the differences became even more apparent, with significantly increased mean concentrations of formic, acetic, propionic, butyric, valeric, isobutyric, and isovaleric acids in females by 61.9%, 19.7%, 40.9%, 22.8%, 34.3%, 51.7%, and 30.6%, respectively (Figure 2A). The opposite was observed in the case of lactic acid, which was lower in the group of females of normal weight by 18.7%.

On the contrary, the gender differences within the group of obese children were noticeably less significant and more diverse (Figure 2B). Concentrations of lactic acetic, propionic, and valeric acids were increased in the male children by 35.9%, 19.4%, and 60.1%, respectively. The opposite was observed for formic, isobutyric, and isovaleric acids, where concentrations were decreased in male children by 12.2%, 21.6%, and 21.6%, respectively. Concentrations of acetic and butyric acids were not significantly different between males and females in the tested group.

### 3.2. Activities of Fecal Enzymes

The aim of this experiment was to evaluate whether the activity of fecal enzymes differed significantly between the group of obese children and the group of children of normal weight. The hypothesis was similar to the one described in the previous section, given that dietary habits and alterations in gut microbiota can potentially influence the enzymatic activity of bacteria in the large intestine. Accordingly, it was suspected that activity of fecal enzymes closely related to the presence of less desired microbiota (associated with obesity) would cause increased activity of potentially harmful enzymes such as β-glucosidase and β-glucuronidase.

**Figure 3:**
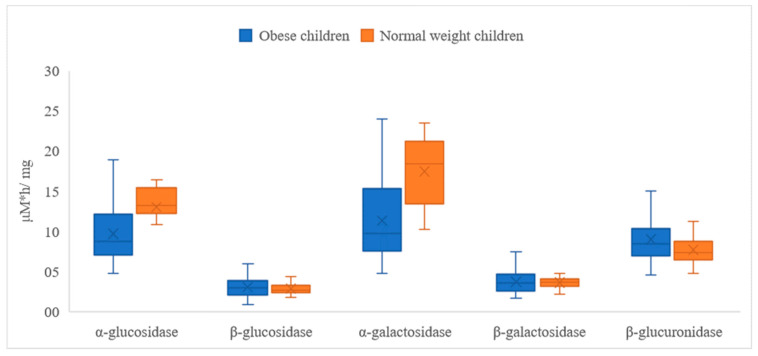
Activity of fecal enzymes in obese versus normal-weight children.

Correspondingly to the concentrations of fatty acids, the calculated activity of fecal enzymes was in most cases significantly different (*p* < 0.05) in the group of children of normal weight when compared to the group of obese children (Figure 3). In the case of α-glucosidase and α-galactosidase, the activity in the obese subjects was, respectively, 25.8% and 35.1% lower than in the case of children with normal weight (Table 4). In the case of potentially harmful enzymes (β-glucosidase and β-glucuronidase), the activity was influenced differently. The activity of β-glucuronidase was significantly higher in the group of obese children (by 16.9%), whereas the activity of β-glucosidase was only slightly lower; however, the result was not statistically different (*p* > 0.05). Similarly, the activity of β-galactosidase was not affected by the BMI of the children (Table 4).

**Figure 4:**
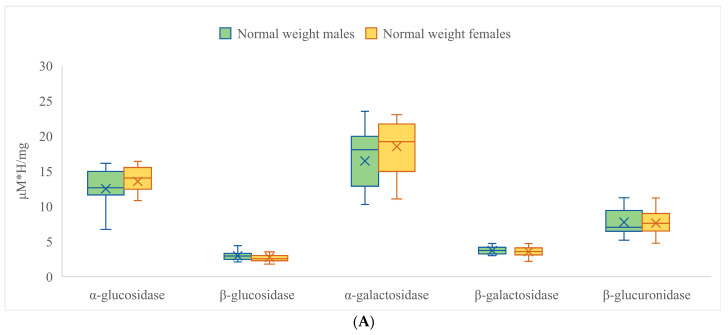

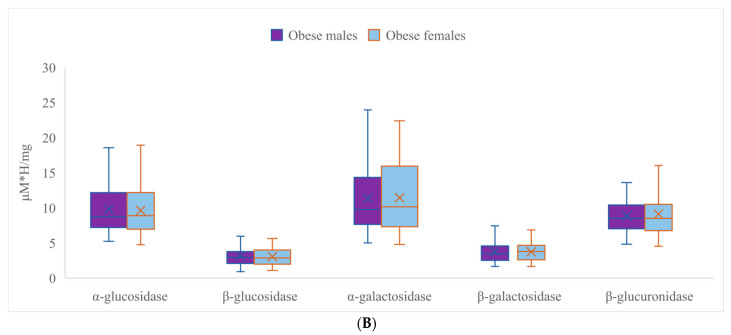
Comparison of the activity of fecal enzymes differentiated between males and females and according to anthropometric status (**A**)—presents data of normal weight males and females; (**B**)—presents data of obese males and females.

Additionally, the comparison of enzymatic activity was conducted between male and female children (Figure 4). Surprisingly, the results for the total population of study participants (displayed in Table 5) demonstrated no significant differences between the groups, which suggests that the relationship between the enzymatic activity and metabolic state of patients is different from in the case of fatty acids. On the other hand, when the same comparison is made between the groups of obese male and female children, and between normal-weight male and female children (Figure 4A,B), it becomes apparent that there are noticeable gender differences in the group of children of normal weight in the case of α-glucosidase and α-galactosidase (both are increased in the female group by 9.2 and 12.8%, respectively). The group of obese children showed no differentiation between genders (Figure 4B).

The mean values for the activity of each enzyme in the group of overweight children and the control group of children of normal weight indicate different patterns of enzyme activity. In the overweight children group, α-glucosidase had the highest mean value (11,453 μMh/mg), followed by α-galactosidase (14,115 μMh/mg) and β-glucuronidase (10,177 μMh/mg). The lowest mean value was observed for β-galactosidase (4103). The ratio of α-glucosidase to β-galactosidase in this group was 2.79:1. In the control group of children of normal weight, α-galactosidase had the highest mean value (17,474 μMh/mg), followed by α-glucosidase (13,015 μMh/mg) and β-glucuronidase (7674 μMh/mg). The lowest mean value was observed for β-glucosidase (2845 μMh/mg). The ratio of α-glucosidase to β-glucosidase in this group was 4.56:1. Comparing the ratios of the enzymes within each group, it appears that the group of overweight children had a lower ratio of α-glucosidase to β-galactosidase compared to the control group. This suggests that the group of overweight children might have had a lower carbohydrate digestion rate and a lower capacity for breaking down galactose, which could contribute to their overweight status. Furthermore, the ratios between the two groups show that the control group had a higher ratio of α-glucosidase to β-glucosidase (4.56:1) compared to the group of overweight children (2.79:1). This indicates that the control group might have had a higher carbohydrate digestion rate, which could help to maintain their normal weight. In the overweight children, the ratio of β-glucuronidase to α-glucosidase was approximately 0.9, while in the control group it was approximately 0.6. This suggests that the activity of β-glucuronidase is relatively higher compared to α-glucosidase in overweight children. Similarly, the ratio of β-glucuronidase to β-glucosidase was approximately 2.9 in the overweight children and 2.7 in the control group, indicating that the activity of β-glucuronidase is also relatively higher compared to β-glucosidase in both groups. In accordance, the ratios of β-glucuronidase to α-galactosidase and β-galactosidase were lower in the overweight children compared to the control group. The ratio of β-glucuronidase to α-galactosidase was approximately 0.7 in the overweight children and 0.6 in the control group, while the ratio of β-glucuronidase to β-galactosidase was approximately 2.5 in the overweight children and 2.1 in the control group.

When comparing the ratios of all enzymes between overweight female children and normal-weight female children (Figure 4A,B), it appears that the normal-weight female children had a higher mean activity for each of the five enzymes compared to the overweight female children. For example, the mean activity of α-glucosidase in normal-weight female children was 13,542 μMh/mg, which was higher than the mean activity of 9625 μMh/mg in overweight female children. This pattern can also be seen in the other enzymes. When comparing the ratios of all enzymes between overweight male children and normal-weight male children, it appears that the normal-weight male children had a higher mean activity for each of the five enzymes compared to the overweight male children. For example, the mean activity of α-glucosidase in normal-weight male children was 12,489 μMh/mg, which was higher than the mean activity of 9770 μMh/mg in overweight male children. When comparing the ratios of α-galactosidase in overweight female children and normal weight female children, it appears that the normal-weight female children had a higher mean activity of the enzyme compared to the overweight female children. The mean activity of α-galactosidase in normal-weight female children was 18,506 μMh/mg, which was higher than the mean activity of 11,425 μMh/mg in overweight female children. When comparing the ratios of β-galactosidase in overweight female children and normal-weight female children, it appears that the normal-weight female children had a higher mean activity of the enzyme compared to the overweight female children. The mean activity of β-galactosidase in normal-weight female children was 3551 μMh/mg, which was lower than the mean activity of 3725 μMh/mg in overweight female children. When comparing the ratios of β-glucuronidase in overweight female children and normal-weight female children, it appears that the normal weight female children had a slightly higher mean activity of the enzyme compared to the overweight female children. The mean activity of β-glucuronidase in normal-weight female children was 7610 μMh/mg, which was lower the mean activity of 9111 μMh/mg in overweight female children. A similar pattern can be seen when comparing the ratios of the enzymes in overweight male children and normal-weight male children. In general, normal-weight male children have higher mean activities of the neutral fecal enzymes and lower activities of potentially harmful fecal enzymes compared to overweight male children.

When comparing the ratios of the five enzymes between male and female children of normal weight (Figure 4A), it appears that male children had lower mean activities of α-glucosidase and β-glucosidase compared to female children. The mean activity of α-glucosidase in male children of normal weight was 12,489 μMh/mg, which was lower than the mean activity of 13,542 μMh/mg in female children of normal weight. Furthermore, the mean activity of β-glucosidase in male children of normal weight was 2953 μMh/mg, which was higher than the mean activity of 2737 μMh/mg in female children of normal weight. However, when it comes to α-galactosidase and β-galactosidase, female children of normal weight had higher mean activities of the enzymes compared to male children of normal weight. The mean activity of α-galactosidase in female children of normal weight was 18,506 μMh/mg, which was higher than the mean activity of 16,442 μMh/mg in male children of normal weight. The mean activity of β-galactosidase in female children of normal weight was 3551 μMh/mg, which was lower than the mean activity of 3705 μMh/mg in male children of normal weight. In terms of β-glucuronidase, there was no significant difference in the mean activity between male and female children of normal weight. The mean activity of β-glucuronidase in male children of normal weight was 7739 μMh/mg, which was only slightly higher than the mean activity of 7610 μMh/mg in female children of normal weight. When comparing the ratios of the five enzymes between overweight male and overweight female children, it appears that there is little difference in mean activities between the two groups (Figure 4B).

### 3.3. Correlation Analysis

**Figure 5:**
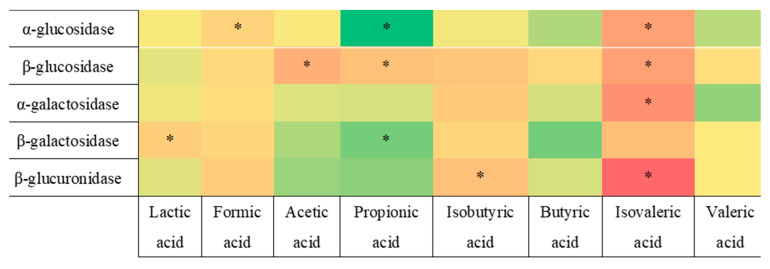
Pearson’s heatmap representing the correlation between the activity of fecal enzymes and concentrations of fatty acids. Green represents negative correlation, whereas orange-red represents positive correlation. *: (*p* < 0.05).

**Figure 6:**
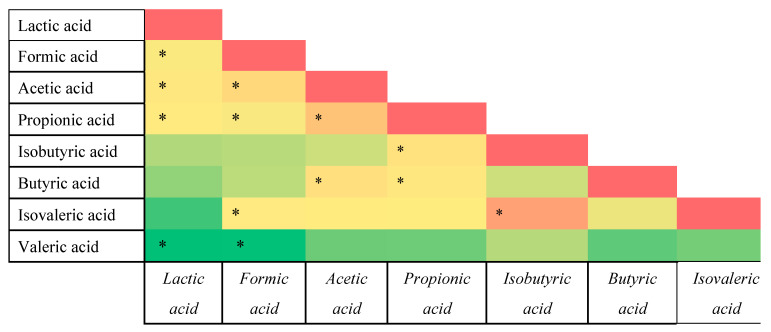
Pearson’s heatmap representing correlation between the concentrations of fatty acids. Green color represents negative correlation, whereas orange-red represents positive correlation. *: (*p* < 0.05).

**Figure 7:**
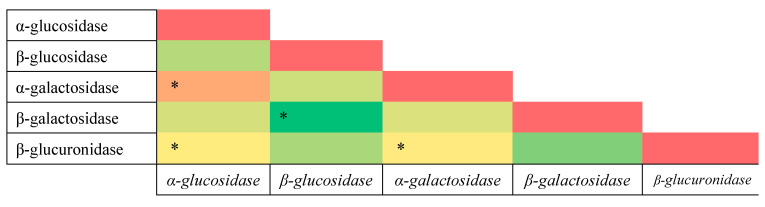
Pearson’s heatmap representing correlation between the activity of fecal enzymes. Green represents negative correlation, whereas orange and red represent positive correlation. *: (*p* < 0.05).

In order to obtain further insight and explore possible trends, different heatmaps were created to determine the possible correlations between tested concentrations of fatty acids and activities of fecal enzymes (Figure 5, Figure 6 and Figure 7).

As demonstrated in Figure 5, the activity of most enzymes has rather insignificant correlations with the concentrations of fatty acids. Even the most relevant correlations discovered in this study (between α-glucosidase and propionic acid, or β-glucuronidase and isovaleric acid) have relatively low values, which suggests that, while they exist, they are not strong.

Significantly stronger correlations were observed between different fatty acids. It was noted that there is relatively strong correlation between SCFAs and equally strong correlation between two tested BCFAs. The strongest correlations were calculated for the concentrations of acetic and propionic acids (Figure 6). Nonetheless, the concentration of lactic acid was also strongly correlated with those of the SCFAs, especially formic, acetic, and propionic acids.

Another strong link was found between the concentrations of isovaleric and isobutyric acids, which further confirms that they are likely produced by the bacteria associated with obesity (Figure 6).

In the case of enzymatic activity in fecal samples, the strongest correlation was discovered between the activity of α-glucosidase and that of α-galactosidase. Surprisingly, there is likewise a correlation between the activity of α-glucosidase, α-galactosidase, and β-glucuronidase (Figure 7.). It is possible that this is because, in normal-weight patients, the general abundance of bacteria was higher, which resulted in a higher count of bacteria producing β-glucuronidase. Nonetheless, the results clearly indicate that, even in this particular case, the activity of α-glucosidase and α-galactosidase was significantly higher in the group of children with normal weight.

In most cases, the outcomes produced may be rationalized by the fact that, due to the BMI disparity, differences in diet and the ratio of probiotic bacteria to less desired genera, the concentrations and/or activity of different metabolites are influenced, favoring either the beneficial or potentially harmful ones. The pH in the large intestine also changes, which can potentially influence the activity of various enzymes. These results support the hypothesis that obesity has significant consequences regarding the production of metabolites in the large intestine of children.

## 4. Discussion

In the presented study, the main objective was to evaluate whether obesity has a significant effect on the metabolites present in the large intestine of children, namely lactic acid, SCFAs (formic, acetic, propionic, butyric, and valeric acid), BCFAs (isobutyric and isovaleric acid), and selected fecal enzymes (α-glucosidase, β-glucosidase, α-galactosidase, β-galactosidase, β-glucuronidase). Additionally, these parameters were also compared by gender.

The obtained results confirmed the stated hypothesis, that obese children have noticeably different activity of fecal enzymes and profiles of fatty acids. The observed disparity is mostly negative, as obese children when compared to children of normal weight had increased concentrations of BCFAs and increased activity of potentially harmful enzymes such as β-glucosidase and β-glucuronidase. Contrastingly, children of normal weight exhibited significantly increased concentrations of lactic acid and SCFAs (especially formic and butyric acids). Nonetheless, there was no linear correlation found between the increase in obesity status (value of BMI) and shifts in the activity of fecal enzymes and concentrations of investigated metabolites. The changes were observed only among two compared groups and among certain individuals, where participants with the highest BMI also exhibited extreme values for tested metabolites. This finding might suggest that other factors are very likely also affecting the state of metabolic activity in the large intestine.

Furthermore, the activity of beneficial fecal enzymes (α-glucosidase and α-galactosidase) was drastically higher when compared to the group of obese children. Such an observation should be considered positive given that these enzymes are beneficial for the host. α-glucosidase allows individuals to digest more fiber, thus producing more SCFAs, while α-galactosidase contributes to the digestion of dairy products which are of high importance for developing children. In comparison, significantly elevated activity of β-glucuronidase may promote the formation of colon cancer [24,25,26]. Moreover, β-glucuronidase (similarly to β-glucosidase) has the ability to convert heterocyclic aromatic amines, polycyclic aromatic hydrocarbons, and some bile acids into harmful compounds (the products of such conversion are carcinogenic aglycons) [27,28,29]. Furthermore, it was found by Li et al. [30] that by inhibition of β-glucosidase it is possible to increase the effectiveness of chemotherapy against colorectal cancer and even to suppress the growth of cancer.

Another important aspect to consider is that, although α-glucosidase is a beneficial fecal enzyme, the α-glucosidase inhibitors are the major antidiabetic agents. A-glucosidase inhibitors are a class of drugs that are used to manage hyperglycemia, or high blood sugar levels, in people with type 2 diabetes. These drugs work by blocking the action of the α-glucosidase enzyme in the gut, which is responsible for breaking down carbohydrates into simple sugars. This results in a slower digestion of carbohydrates and a slower release of glucose into the bloodstream. Studies have shown that α-glucosidase inhibitors can have an effect on the gut microbiota and fecal enzyme activity [31,32,33]. In general, these drugs have been shown to cause alterations in the gut microbial community, including changes in the abundance of certain bacterial species and the overall diversity of the gut microbiome. In terms of fecal enzyme activity, α-glucosidase inhibitors can decrease the production of certain enzymes, including α-glucosidase, which is the target of these drugs. This decrease in enzyme activity can affect the ability of the gut microbiome to digest and utilize carbohydrates, potentially leading to changes in the composition of the gut microbiota. It is important to note that the effects of α-glucosidase inhibitors on the gut microbiota and fecal enzyme activity may vary between individuals, and more research is needed to fully understand the implications of these changes. Additionally, the effects of these drugs on the gut microbiome may depend on the dose and duration of treatment, as well as other factors such as diet and lifestyle. Overall, the effects of α-glucosidase inhibitors on the gut microbiota and fecal enzyme activity are an area of active research, and it is important to monitor and understand these effects in order to optimize the use of these drugs in managing hyperglycemia in type 2 diabetes. Further studies are needed to determine the long-term implications of these changes on human health, and to develop strategies for mitigating any negative effects.

It is also important to note that elevated glucose levels in the bloodstream can contribute to the development of insulin resistance, which is a hallmark of type 2 diabetes [34]. High α-glucosidase and β-glucosidase activity can increase glucose levels by breaking down complex carbohydrates into simple sugars that are rapidly absorbed into the bloodstream. In response to elevated glucose levels, the pancreas secretes insulin to bring glucose levels back to normal. However, if this process occurs repeatedly over time, the cells in the body may become resistant to insulin, leading to higher glucose levels that are harder to control [35]. Another important aspect to consider is the role of incretins in regulating glucose levels. Incretins are hormones that are released in response to food intake and stimulate insulin secretion to help regulate glucose homeostasis by increasing insulin secretion and decreasing glucagon secretion in response to a meal. High α-glucosidase activity can increase incretin secretion and enhance insulin secretion, leading to improved glucose control [36]. This enzyme is also important for the metabolism of dietary fibers, which are indigestible carbohydrates that are fermented in the large intestine by the gut microbiota. Increased activity of these enzymes can lead to an increase in the production of short-chain fatty acids (SCFAs), which can stimulate the release of incretins such as glucagon-like peptide 1 (GLP-1) and glucose-dependent insulinotropic polypeptide (GIP) [37,38].

This highlights the interplay between fecal enzyme activity, glucose levels, insulin secretion, and incretin levels, and underscores the importance of considering all these factors when trying to understand and manage glucose metabolism. It is also worth noting that fecal enzyme activity can vary between individuals and can be influenced by a variety of factors, including diet, genetics, and gut microbiome composition. For example, diets high in complex carbohydrates can increase the demand for α-glucosidase and β-glucosidase, while diets high in simple sugars can lead to decreased demand for these enzymes. Understanding the factors that influence fecal enzyme activity can help us better understand the factors that contribute to glucose metabolism and inform the development of new therapeutic approaches for metabolic disorders. In summary, the effects of fecal enzyme activity on glucose, insulin, and incretin levels are complex and multifaceted, and a better understanding of these effects can induce the development of new approaches to manage glucose metabolism and metabolic disorders.

An interesting finding was discovered after a comparison of profiles of metabolites by gender. These results clearly indicated that fatty acids were affected by gender, in several cases by a significant amount in both groups of children (obese and with healthy weight). Nonetheless, this phenomenon was observed to a lesser extent in the case of the activity of fecal enzymes, where differences between genders were found only within group of children of normal weight.

Furthermore, the in-depth analysis of the results suggested that there may be some differences in the ratios of fecal enzymes between male and female children, with male children generally having higher mean activities of α-glucosidase and β-glucosidase, while female children generally having higher mean activities of α-galactosidase and β-galactosidase. However, these differences may not be significant, and further research is needed to fully understand the impact of gender on fecal enzyme activities.

Nonetheless, when comparing the ratios of the enzyme activities within each group, it is important to keep in mind that the ratios will vary depending on the specific activity of each enzyme. The ratios can provide insight into the relative proportions of the different enzymes present in each group, but they should not be used to make direct comparisons between the groups. When comparing the enzyme activity ratios between the two groups, it is important to note that the control group had higher levels of all five enzymes studied, which suggests that the gut microbiota in the control group may be more diverse and healthier compared to the gut microbiota in the overweight group. In conclusion, the results of this study suggest that there are differences in the gut microbiota and fecal enzyme activity levels between overweight children and children of normal weight, but further research is needed to better understand the underlying mechanisms and the potential implications of these differences for health and disease.

All these differences can be explained by shifts of balance in the gut microbiota or changes in its diversity caused by unhealthy (or gender-influenced) diet or associated with the occurrence of obesity, which can have a major impact on the secretion of active compounds such as lactic acid, SCFAs, and BCFAs and the activity of fecal enzymes [14,39,40]. However, further studies on the subject are necessary to assess the differences in dietary habits between children of different genders, and to link these habits with the occurrence of specific strains or makeups of microbiota present in the large intestine. It may be suspected that specific genders, even at young age, tend to follow a different dietary trend either imposed by parents, society, or their own preferences, which finally lead to differences in microbiota composition. Another factor might be connected with hormonal development or differences in children, which may also influence the composition of fecal microbiota, which was demonstrated in several studies [41,42,43].

Fatty acids are produced in the large intestine as byproducts of the fermentation of dietary fibers and resistant starches by several species of bacteria residing in this environment. As mentioned before, SCFAs have many confirmed positive effects on the human body, while BCFAs are instead associated with negative impacts. Nonetheless, many studies have shown their beneficial effects, i.e., anti-inflammatory, anticarcinogenic, and anti-obesity, which suggests that their concentration might play a crucial role in the properties they exhibit [44,45].

Even though the topic of links between the anthropometric status of patients and the metabolites of their microbiota has been raised in the scientific community, to the author’s current knowledge there are not many studies that directly compare these differences. An interesting study by Covian et al. [46] revealed quite similar findings to our study. It was shown that concentrations of BCFAs (isobutyrate and isovalerate) were directly correlated with the BMI. The higher the BMI, the higher the BCFA concentration. Nonetheless, contrary to our findings, the overall effect on fatty acids in the above-mentioned study was considered non-significant.

Moreover, several studies have indicated the association of specific genera of gut bacteria with either health benefits or the development of diseases. Bacteria from genera Clostridium, Bacteroides, Enterococcus, and Escherichia are usually presented as non-desired microbiota and are related to increased activity of β-glucuronidase (EC 3.2.1.31) and β-glucosidase (EC 3.2.1.21), which both have a significant correlation with the development of colorectal cancer [27,47,48]. In contrast, genera such as Bifidobacterium and Lactobacillus present a positive impact on the enzymatic activity in the gastrointestinal tract. Various studies have demonstrated their anticarcinogenic properties and ability to decrease the activity of carcinogen-metabolizing enzymes [49,50]. First and foremost, these species are also directly connected with the prevalence of obesity. Bacteria from the genera Clostridium, Bacteroides, Enterococcus, and Escherichia are more prevalent in obese patients, while species from the genera Bifidobacterium and Lactobacillus are often considered probiotics and are associated with a healthy and balanced diet rich in fibers and vitamins [8,51,52].

Furthermore, the gut microbiota and its metabolites might be affected by various other factors such as the amount and bioavailability of amino acids in the intestines, different metabolic pathways not necessarily directly linked to the gut, or several diseases such as depression and anorexia [46,53,54,55]. Hence, the studied metabolites can be treated as health markers, but with serious consideration and caution.

Further limitations of this study include that the determination of metabolites, both fatty acids and fecal enzymes, was performed in fecal samples, which does not necessarily reflect the actual concentrations and activity of these metabolites in different parts of the colon, but are mainly derived from the end of the gastrointestinal tract. Additionally, the tested metabolites are in constant fluctuation due to their absorption in the large intestine. Therefore, the true concentrations or activities of these metabolites might vary slightly. A further important factor that was not considered in this study was the water content of feces, which could also influence the results. Nonetheless, this problem was partially prevented by the selection process in which children with gastric problems were excluded from the study.

In light of these findings and the limitations, further studies on the comparison of profiles of metabolites produced by the gut microbiota in patients with different anthropometric statuses should be conducted to acquire further insight in the field of relationships in the gastrointestinal environment of humans.

## 5. Conclusions

In conclusion, the difference between the investigated metabolites present in the fecal samples of children with obesity and with healthy BMIs is considerable. Obese children exhibited significantly higher concentrations of BCFAs, which are associated with gut dysbiosis. Moreover, the results showed that their levels of lactic acid and SCFAs were lower in comparison with the children of healthy weight. Similar findings concerned the activity of fecal enzymes. The activity of beneficial α-glucosidase and α-galactosidase was significantly higher in the group of children of normal weight and lower in the obese children. In comparison, the activity of β-glucuronidase (similarly to β-glucosidase) increased in the group of children with higher BMI. These findings further confirm the negative effects linked with the prevalence of obesity, which can lead to the development of severe diseases.

Interestingly, differences were found in the profiles of fatty acids with gender as the division factor. This might designate the direction for future research on either the social level of diet composition for different genders, or on possible differences in the composition of the microbiota. However, gender had no significant implications for the activity of fecal enzymes.

Further studies are necessary to shed more light on the vast network of interactions between the gut microbiota, their environment, and anthropometric factors of humans.

## Figures and Tables

**Table 1:** Characterization of the participants of the study.

	Participants of the Study			
	Overweight and Obese Children		Normal-Weight Children	
Gender	Male	Female	Male	Female
	46	51	13	13
Age [years]	8.67 ± 1.05	8.26 ± 1.33	7.85 ± 1.65	7.77 ± 1.57
Weight [kg]	51.55 ± 11.16	44.53 ± 10.76	26.27 ± 6.97	25.23 ± 5.02
Height [m]	1.43 ± 0.10	1.37 ± 0.10	1.35 ± 0.14	1.32 ± 0.13
BMI [kg·m^−2^]	25.06 ± 3.67	23.49 ± 3.45	14.04 ± 1.94	14.36 ± 1.25

The table contains average values with standard deviation.

**Table 2:** Concentrations of lactic acid, SCFAs, and BCFAs in obese children and children of normal weight.

	Lactic Acid		Formic Acid		Acetic Acid		Propionic Acid	
	Obese	Normal Weight	Obese	Normal Weight	Obese	Normal Weight	Obese	Normal Weight
Mean	2.877 *	3.840 **	2.007 *	2.759 **	3.743 *	4.285 **	1.648 *	1.877 **
SD	3.401	3.334	1.870	1.293	1.452	1.394	1.105	0.803
Range	20.483	10.345	9.814	5.257	7.959	5.513	6.980	3.364
Min	0.142	0.673	0.116	0.273	0.027	1.001	0.008	0.410
Max	20,625	11.018	9.929	5.531	7.987	6.514	6.988	3.774
	**Butyric acid**		**Valeric acid**		**Isobutyric acid**		**Isovaleric acid**	
	**Obese**	**Normal weight**	**Obese**	**Normal weight**	**Obese**	**Normal weight**	**Obese**	**Normal weight**
Mean	1.651 *	2.327 **	1.796	1.998	0.830 *	0.703 **	0.572	0.524
SD	1.757	1.124	2.436	1.727	0.572	0.475	0.302	0.224
Range	15.127	4.710	15.718	6.535	2.025	1.645	1.238	1.000
Min	0.000	0.666	0.000	0.069	0.000	0.024	0.000	0.033
Max	15.127	5.376	15.718	6.605	2.025	1.669	1.238	1.034

The mean is the result calculated from three repetitions for each of 123 samples. The results are significantly different from: *—the children of normal weight; **—the obese children; one-way ANOVA with post-hoc Tukey’s test (*p* < 0.05). SD: Standard deviation; Unit: mg/g.

**Table 3:** Concentrations of lactic acid, SCFA and BCFA in the group of males and females.

	Lactic Acid		Formic Acid		Acetic Acid		Propionic Acid	
	Male	Female	Male	Female	Male	Female	Male	Female
Mean	3.528 *	2.672 **	1.935 *	2.373 **	3.843	3.869	1.750	1.647
SD	3.520	3.200	1.394	2.045	1.444	1.446	1.229	0.843
Range	18.312	20.483	6.805	9.747	5.978	7.959	6.980	3.568
Min	0.517	0.142	0.116	0.182	1.001	0.027	0.008	0.206
Max	18.829	20.625	6.921	9.929	6.979	7.987	6.988	3.774
	**Butyric acid**		**Valeric acid**		**Isobutyric acid**		**Isovaleric acid**	
	**Male**	**Female**	**Male**	**Female**	**Male**	**Female**	**Male**	**Female**
Mean	1.713	1.865	2.128 *	1.575 **	0.703 *	0.894 **	0.500 *	0.618 **
SD	1.155	2.003	2.704	1.799	0.531	0.554	0.284	0.277
Range	5.041	15.127	15.718	8.332	1.995	2.023	1.147	1.231
Min	0.086	0.000	0.000	0.000	0.000	0.001	0.000	0.007
Max	5.127	15.127	15.718	8.332	1.995	2.025	1.147	1.238

The mean is the result calculated from three repetitions for each of 123 samples. The results are significantly different from: *—females; **—males; one-way ANOVA with post-hoc Tukey’s test (*p* < 0.05). SD: Standard deviation; Unit: mg/g.

**Table 4:** Comparison of the activity of fecal enzymes in the group of obese children versus the group of children of normal weight.

	α-Glucosidase		β-Glucosidase		α-Galactosidase	
	Obese	Normal Weight	Obese	Normal Weight	Obese	Normal Weight
Mean	9.660 *	13.015 **	3.077	2.845	11.348 *	17.474 **
SD	3.373	2.810	1.289	0.759	5.004	4.124
Range	14.175	9.772	7.848	3.410	19.160	13.246
Min	4.745	6.621	0.913	1.779	4.780	10.258
Max	18.920	16.393	8.760	5.190	23.941	23.504
	**β-galactosidase**		**β-glucuronidase**			
	**Obese**	**Normal weight**	**Obese**	**Normal weight**		
Mean	3.718	3.628	8.975 *	7.674 **		
SD	1.303	0.651	2.917	1.825		
Range	5.786	2.529	12.981	6.464		
Min	1.645	2.176	4.516	4.743		
Max	7.430	4.705	17.497	11.207		

The mean is the result calculated from three repetitions for each of 123 samples. The results are significantly different from: *—the children of normal weight; **—the obese children; one-way ANOVA with post-hoc Tukey’s test (*p* < 0.05). SD: Standard deviation; Unit: μMh/mg.

**Table 5:** Comparison of activities of fecal enzymes in the group of males versus females.

	α-Glucosidase		β-Glucosidase		α-Galactosidase	
	Male	Female	Male	Female	Male	Female
Mean	10.369	10.408	3.093	2.987	12.467	12.842
SD	3.507	3.514	1.293	1.097	5.376	5.430
Range	13.336	14.175	7.848	4.555	18.940	18.242
Min	5.231	4.745	0.913	1.078	5.000	4.780
Max	18.567	18.920	8.760	5.633	23.941	23.022
	**β-galactosidase**		**β-glucuronidase**			
	**Male**	**Female**	**Male**	**Female**		
Mean	3.731	3.690	8.614	8.811		
SD	1.215	1.167	2.556	2.943		
Range	5.781	5.206	11.092	12.981		
Min	1.650	1.645	4.815	4.516		
Max	7.430	6.851	15.907	17.497		

The mean is the result calculated from three repetitions for each of 123 samples.

## Data Availability

All data used in this study are freely accessible to the public via the papers used for research, which are referenced in the bibliography.The corresponding authors K.Ś. and M.W. also provide access to the data used in this investigation.

## Abbreviations

SCFA	short-chain fatty acid
BCFA	branched-chain fatty acid
